# Improvement of Drug Delivery Properties of Risperidone via Preparation of Fast Dissolution Tablet Containing Nanostructured Microparticles

**DOI:** 10.22037/ijpr.2020.112230.13621

**Published:** 2021

**Authors:** Motahareh Salarvand, Vahid Ramezani, Fatemeh Salarvand, Zeinab Aref Darabi, Maryam Akrami

**Affiliations:** a *Department of Pharmaceutics, Faculty of Pharmacy, Shahid Sadoughi University of Medical Sciences, Yazd, Iran.*; b *Department of Psychiatry, Faculty of Medicine, Arak’s University of Medical Sciences, Arak, Iran. *; c *Department of Psychiatry, Faculty of Medicine, Shahid Sadoughi University of Medical Sciences, Yazd, Iran.*

**Keywords:** Nanoparticles, Dissolution, Risperidone, Fast dissolution tablet, Spray freeze-drying

## Abstract

Aimed to improve the dissolution profile of risperidone and increase the compliance of psychotic patients, we designed a fast dissolution tablet (FDT) containing nanoparticles. Risperidone nanoparticles were prepared by the acid-alkali neutralization method, and their size and stability were evaluated. Spray freeze-drying (SFD) process was then employed to fabricate the nanoaggregates using sugars. The physicochemical properties of the dried powders were assessed. Finally, nanoaggregates were compressed into tablets, and their properties were evaluated. The results show that the synergic effect of cremophore EL and hydroxypropyl methyl cellulose E_15_ can give rise to the formation of risperidone nanosuspension with the particle size of 188 nm. Moreoevr, it is shown that the fabrication of risperidone nanoaggregate enhanced the drug dissolution and decreased that to 2 min, which is faster than coarse risperidone powder (with dissolution time of 60 min). The formulations of FDT containing 9.5% of sodium starch glycolate and 83.2% microcrystalline cellulose were selected with a disintegration time of less than 30 s and a dissolution time of 10 min. This investigation shows that the preparation of FDT containing nanoparticles using SFD is an easy and feasible method for improving the dissolution profile of many drugs with low solubility.

## Introduction

The oral drug delivery system is one of the safe‚ easy, and economic approaches for drug administration. Many advanced technologies have been engaged to introduce tablets as a common dosage form with high patient compliance (1). Amongst them, FDT is capable of fast delivery of the active substance in the oral cavity. Also, they are suitable for pediatric‚ geriatric‚ mental retard and psychotic patients as well as patients unable to swallow a tablet or refuse that. On the other hand, according to the high susceptibility of drug absorption in the pregastric zone (such as mouth‚ pharynx and esophagus), the FDTs are very suitable to enhance the drug absorption (2-4). Therefore, the bioavailability of drugs can be enhanced by avoiding the first-pass effect to increase the clinical effect (5). However, it should be noticed that the active substant must be dissolved after disintegration, which is the limitation of FDT for low soluble drugs.

The poor bioavailability of the drug in class II biopharmaceutical system is due to dissolution rate limitations (6, 7), which was resolved in some studies by increasing the surface area of drug particles according to Noyes Whitney equation (8). Any decrease in the size of nano-scaled particles shall increase its dissolution rate up to 40-fold (9). Therefore the particle size engineering is one of the dominant ways to enhance the dissolution rate of low soluble drugs (10). In this way, nanosuspension technology has played a key role in dissolution enhancement. This process is carried out by two main approaches; bottom-up and top-down. In the bottom-up approach, the particle size reduction is obtained by controlling the precipitation/crystallization. In the top-down approach, the particle size is reduced by mechanical attrition of large crystalline particles into nanoparticles (9, 11-13).

High inter-particulate adhesion is the main problem in the manufacturing and processing the nanoparticle. These problems include low flowability and high affinity for the accumulation and aggregation (14). One way to overcome these obstacles is to change the microparticles (15-17). Nowadays, some processes such as spray drying, spray freeze-drying, and freeze-drying are employed to prepare microparticles (18). Although spray drying is one of the conventional methods for the preparation of dry powder, some disadvantages such as high temperature and instability of labile drugs have limited its application (6, 15, 18). In the other hand, spray freeze-drying (SFD) is one of the suitable technologies to dry the nanosuspension without dramatic instability (14, 15).

Risperidone is one of the atypical antipsychotic which acts by blocking the serotonin (5HT2A) and dopamine (D_2_) receptor (19). However, this agent is classified into the biopharmaceutical classification system (BCS) II because of low solubility (aqueous solubility at 25 ºC and 37 ºC is 2. 8 and 5 µg/mL, respectively). Therefore, even a slight increase in the dissolution rate of risperidone can greatly increase its bioavailability (20, 21). To improve the dissolution rate of risperidone, various types of methods including preparation of solid dispersion and complexing with resin or cyclodextrins have been suggested (22, 23).

Aiming to improve the dissolution rate and enhance the absorption of risperidone, we fabricated the microparticles containing risperidone nanosuspension and investigated the effect of different surfactants in the preparation of nanoparticles. Spray freeze-drying was applied to fabricate the nanoaggregate powder. Finally, fast dissolution tablets (FDT) were produced and evaluated.

## Experimental

*Materials*

Risperidone USP (RIS) was supplied by Sobhan Darou Co. Cremophor EL and Poly vinyl pyrrolidone (PVP, MW: 10000) were purchased from Sigma (Germany), and sodium Carboxymethyl cellulose (CMC) was supplied from KBR company (USA). Hydroxypropyl methyl cellulose (HPMC E15; 15 cP) was provided from JRS Pharma (Germany) and, D (−) Mannitol and lactose were obtained from Sigma (USA). Hydroxypropyl methyl cellulose (HPMC K4M; 3500–5600 cP) was provided from Colorcon®, Dow Chemical Co (USA). Polyoxyethylene 20-sorbitan monooleate (Tween 80, polysorbate 80) and other excipient used in the preparation of FDT including, microcrystalline cellulose (MCC), silicon dioxide (Aerosil), magnesium stearate, and sodium starch glycolate (SSG) were all obtained from Merck (Germany).

*Methods*

*Preparation of risperidone nanosuspensions*

The initial nanosuspensions were prepared using an acid-alkali neutralization method (9). In the beginning, 10 mg of risperidone was dissolved in 5 mL of deionized water (which contained different types of surfactants or polymeric stabilizers) followed by the addition of HCl aqueous solution (10%, w/w) as mentioned in Table 1 (9, 24). NaOH aqueous solution (1N) was then added to the solution under agitation (250 rpm) until risperidone nanoparticles were formed. All of the formulations were evaluated, and the one with smaller and more stable particles was selected.

*Spray freeze-drying of nanosuspension*

Aqueous nanosuspension was dried with spray freeze-drying with the aid of matrix-forming agents, according to Table 2. Mannitol, lactose, and maltodextrin were added to risperidone aqueous solution before nanoparticle formation. SFD was performed with the spraying of nanosuspension with a rate of 1.8 ml/min in a vessel containing 400 mL of liquid nitrogen followed by lyophilization of the frozen droplets after evaporation of the liquid nitrogen. The frozen droplet was dried with lyophilization for 24 h in a lab-scale freeze-dryer (Christ, Germany) at −55 °C and a vacuum level of 0.05 mbar.

*Nanoparticle size and zeta potential analysis*

The mean particle size and polydispersity index of different risperidone nanoparticles were determined by nanoparticle size analyzer (Brookhaven/90Plus/BI-MASS). Prior to measurements, nanosuspensions were diluted or dried powder was redispersed in saturated RIS solution followed by vortex shaking and sonication for 3 min. The zeta potential was measured by the Malvern Zetasizer Nano® instrument (Malvern, UK).

*Micromeritics properties*

The obtained powders were analyzed for flowability and compressibility parameters, such as tap density (p_tap_), balk density (p_bulk_), Carr’s index (CI), and Hausner̕s ratio with following equations (Wang *et al. *2012):

Carr’sindex (CI) = ($\frac{\rho_{\mathrm{tap}}-\rho_{\mathrm{bulk}}}{\rho_{\mathrm{tap}}})\times 100$

*Scanning electron microscopy*

The samples were mounted onto an aluminum stub and sputter-coated for the 90s with gold-palladium (Bal-Tec, Germany) before surface morphology study by scanning electron microscope (Philips, Netherlands).

*Fourier transform infrared spectroscopy (FT-IR)*

Fourier transform infrared spectroscopy using a Nicolet Magna spectrometer (USA) was employed to evaluate the interaction or complexation of risperidone and other additives. The samples were grounded and mixed thoroughly with potassium bromide to make the clear window, and the spectrum was evaluated between the wave number of 4000- 400 cm^-1^.

*Differential scanning calorimetry (DSC)*

A differential scanning calorimeter (Mettler-Toledo, Switzerland) was used to analyze the thermal behavior of samples. In brief, 5 mg of sample was placed in aluminum pans and heated at the rate of 10 ºC/min from 10 ºC to 310 ºC.

*Preparation risperidone fast dissolution tablet*

The fast dissolution tablets with various formulations were prepared by the direct compression method. All the ingredients were weighed and passed through #40 mesh separately and compressed using a 6.5 mm size flat-faced punch on a single punch compression machine (EP-1, ERWEKA, Germany) to obtain tablets with a weight of 150 mg and hardness of 2 ± 0.5 kg/cm^2^. Then the disintegration time of various formulations was measured, and the most optimized formulation was selected.

*Evaluation parameters of fast dissolving tablets*

*In-vitro disintegration test*

The disintegration time of tablets was tested using the tablet disintegration test apparatus (ZT 221, ERWEKA, Germany). The test was performed in phosphate buffer (pH 6.8) at 37± 2 °C. The time taken for the complete disintegration of the tablet was measured in seconds.

*In-vitro dissolution profile*

*In-vitro* dissolution study of the tablets and powder of nanoparticles was performed using USP dissolution test apparatus-II (paddle assembly). The dissolution was performed using 20 ml SSF (simulated saliva fluid) with a pH of 6.8 as dissolution mediums maintained at 37 ± 0.5 °C with an agitation rate of 50 rpm (25). Aliquots of the dissolution medium (3 mL) were withdrawn at specific time intervals and replaced immediately with an equal volume of fresh medium. Samples were filtered through a syringe filter and assayed spectrophotometrically on the UV-VISIBLE spectrophotometer at 284 nm.

*Statistical Analysis*

To compare different formulations, statistical analysis was determined using the similarity factor (f2) that is a logarithmic reciprocal square root transformation of the sum of squared error and is a measurement of the similarity in the percent (%) dissolution between the two curves. The dissolution profiles were considered similar when f2 was in the range of 50 to 100, and an average difference of 10% at all measured time points results in a f2 value of 50. It was calculated by the following formula:

f_2 =50.log{${\left[1+\frac{1}{n}\sum_{t=1}^{n}{\left(R_{t}-T_{t}\right)}^{2}\right]}^{-0.5}$× 100}

Where n is the number of the sampling time, and Rt and Tt are the references and test dissolution values at time t, respectively (26, 27).

## Results and Discussion

*Preparation of risperidone nanosuspension*

Risperidone nanosuspension was prepared by acid-alkali neutralization technique. In this regard, various types of surface-active agents or polymeric stabilizers were analyzed in nanoparticle formation and stabilization to obtain the nanosuspension with favorable particle size and size distribution. According to Table 1, various types of stabilizers such as ionic surfactant (NaCMC), nonionic surfactants (polysorbate80, Cremophor EL) and semi-synthetic polymers (PVP, HPMC, HPMC E_15_) were investigated in various ratios.

**Figure 1:**
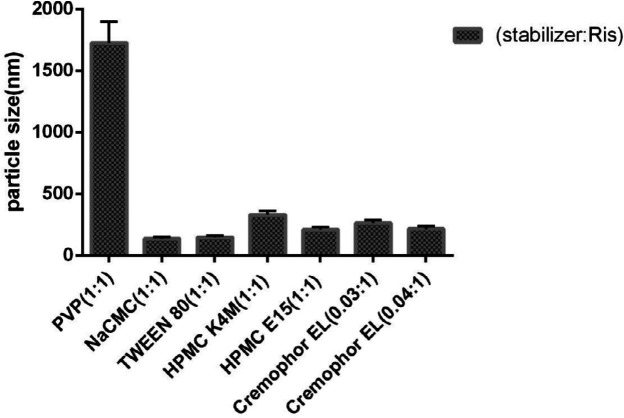
particle size of nanoparticles using different surfactant or polymeric stabilizers in 1:1 ratio (surfactant/RIS).

As shown in Figure 1, various types of stabilizers with different ratios were employed to produce risperidone suspension in the nano-sized range. Among them, ionic NaCMC and nonionic tween 80 surfactants were more efficient in producing smaller nanoparticles (135 ± 1.073 nm and 145 ± 1.073 nm, respectively). However, the application of nonionic polymeric stabilizers such as HPMC K4M and HPMC E_15 _resulted in larger particles with sizes of 209 ± 1.073 nm and 328 ± 1.073 nm, respectively. In the same way, the use of 1:1 PVP/drug as a polymeric stabilizer led to the formation of suspensions with large agglomerated particles (1724 ± 1.073 nm). On the other hand, Cremophor El, as a non-ionic surfactant, in the ratio of 0.04:1, reduced the size of the nanoparticles to 214 ± 1.073 nm.

**Figure 2:**
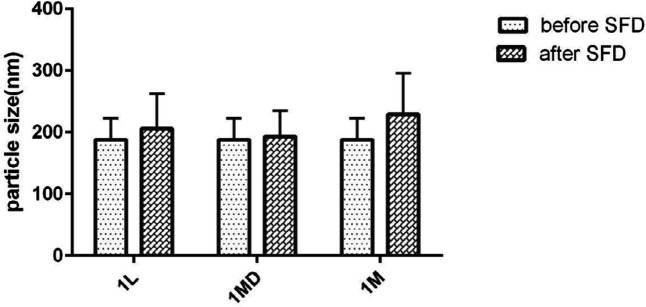
Particle size of nanoparticles before and after SFD

Evaluation of the particle stability utilizing particle size analyzer as well as microscopic observation showed that risperidone nanoparticles were prone to particle growth some minute after preparation, as the risperidone particle size grew to 1 µm in formulations containing nonionic and anionic stabilizers because of susceptibility to Ostwald ripening and crystal growth. However simultaneous usage of HPMC E_15_ and Cremophor EL (with the ratio of 3:0.04:1 to the drug) was suitable to produce the nanoparticles with the size of 188 ± 1.511. It strongly stabilized the nanosuspension without significant size increase after SFD (Figure 2). In general, zeta potential of particles should be at least −20 mV for sterically stabilized systems to obtain a physically stable nanosuspension (28). The zeta potential of optimized nanosuspension was found to be −8.43 ± 0.00 mV, indicating that the prepared nanosuspension do not suffer from instability problems.

**Figure 3:**
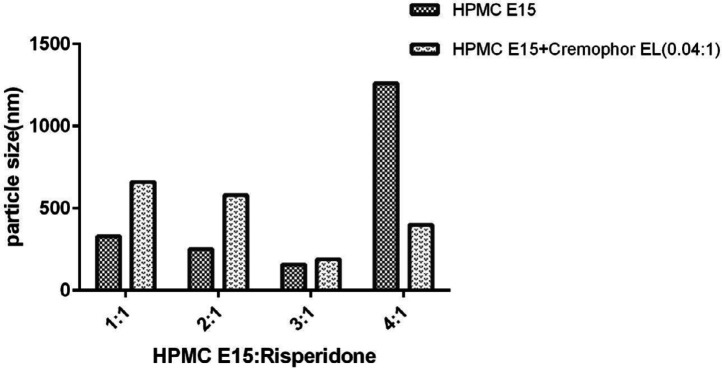
Particle size of nanoparticles using HPMC E15 alone and in combination with Cremophore EL

In general, the process of nanoparticle stabilization is dependent on several factors, including the interaction between drug and stabilizer, the viscosity of medium and the amount of drug surface energy. On the other hand, the nature of surfactants plays a significant role in forming efficient and stable nanoparticles (10, 29). Therefore, there is not a unique method to select the optimal stabiliser for nanosuspension preparation. Surfactants can stabilize nanoparticles by reducing surface activation energy (30). This process involves two major mechanisms: steric stability (often for nonionic surfactants and polymers) and electrostatic stability (for anionic or cationic surfactants) (29). As a hydrophilic polymer, HPMC E_15_ can prevent particles from being aggregated by creating a steric barrier around the particles. Moreover, adsorption of HPMC E_15_ on the surface of the particles will reduce the surface interfacial energy of nanoparticle and will stabilize them. Since this method does not involve drug encapsulation, the drug loading is 100% (9). The results also showed that particles’ size decreased with the increase of surfactant concentration up to 0.6% (w/v). However, further increase in HPMC concentration showed the opposite results (Figure 3). As demonstrated by other studies, freeze-drying can induce aggregation in nanoparticles according to the generation of new surface such as ice crystal surface in the medium (31). Therefore, the migration of surfactant to the new surface can decrease their efficacy and induce particle aggregation especially in a lower concentration of surfactant. In a higher amount of HPMC E_15_, the nanoparticles’ size increased because of enhanced nanosuspension viscosity (32).

**Figure 4:**
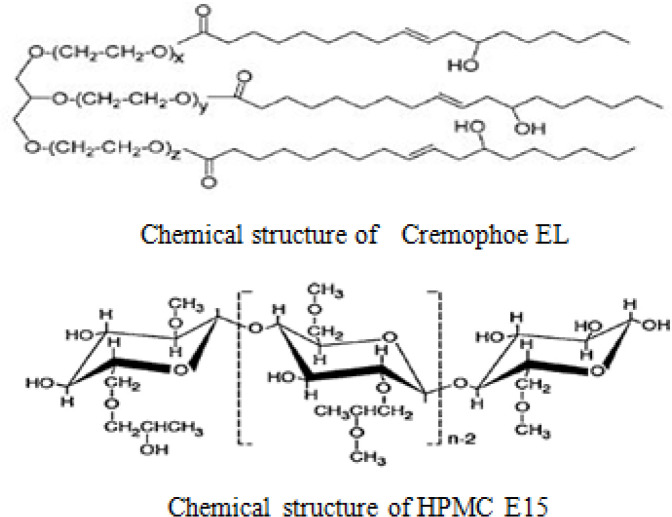
Chemical structure of Cremophor EL and HPMC E15

There are a few reports on the development of drug nanoparticles by using Cremophor EL. This surfactant is prepared by reacting castor oil with ethylene oxide (33). Previous studies have shown that nonionic nonpolymeric surfactant such as Cremophor EL, has a higher adsorption potential on the particles’ surface compared with the polymer of the same length (10). The steric barrier of Cremophor EL and interaction with a lipophilic part of risperidone will cover the nuclei more efficiently and create an inhibitory effect. It will also reduce the inter nuclei interaction and prevent particle growth (Figure 4). Further increase in the concentration of Cremophor EL from 0.006 to 0.008% (w/v) has a beneficial effect on particle size reduction (Figure 1). Moreover, the application of Cremophor EL and HPMC E_15 _(formulation A_12_) was appropriate to increase the stability in the size of particles for further studies.

*Particle size of nanoparticles after SFD*

As suggested by Figure 2, the size of the nanoparticles was not influenced after SFD. This means that the application of sugars as the soluble carriers can preserve the nanoparticles from aggregation and maintain the physical stability of particles. As shown in Figure 2, the nanoparticle preservation ability of lactose and maltodextrin was higher than mannitol. However, the formulation containing mannitol as a matrix agent exhibited less redispersibility with a larger mean particle size. In addition, the results showed that PDI was between 0.1-0.3 reflecting mid-range polydispersity in all the formulations (data not shown) (11).

Generally, the transformation of nanoparticles to dry powder can reduce their physical, chemical, and microbial instability and will make them suitable for the preparation of solid dosage forms (34, 35). Spray freeze-drying is a well-known method to increase the stability of sensitive nanoparticles (15, 17, 36 and 37). Although, spray drying is the more common method to prepare the powder some disadvantages such as high temperature and instability of labile drugs have limited its application (6, 15 and 18). Two important factors determine the particle size and morphology of nanoparticles in the spray drying: evaporation rate and solute diffusion. The high evaporation rate of the droplet and low diffusion coefficient of nanosuspension leads to the accumulation of particles in the droplet surface and its instability. The use of SFD will reduce the aggregation of the nanoparticles by the rapid freezing of the substances and preventing further diffusion of nanoparticles and formation of irreversible aggregated. In addition, due to the low temperature of drying, the physical and chemical damage to nanoparticles will be minimum (3, 18 and 38).

For this purpose, we used mannitol, lactose, and maltodextrin as matrix agents and cryoprotectants to preserve nanosuspension. In General, cryoprotectants prevent irreversible aggregation of the nanoparticle by filling the nanoparticle gaps created during water sublimation at the drying stage (35, 38). Maintaining the size of nanoparticles was achieved by sugar and its high redispersibility which carried out via different mechanisms including; maintaining the glassy state of sugars during freezing and prevention of ice nucleation and mechanical stress caused by ice crystals (which can lead to the formation of irreversible coalescences in nanoparticles) and coating the nanoparticles with sugars and prevention of direct contact during SFD (15, 36).

*SEM test*

**Figure 5:**
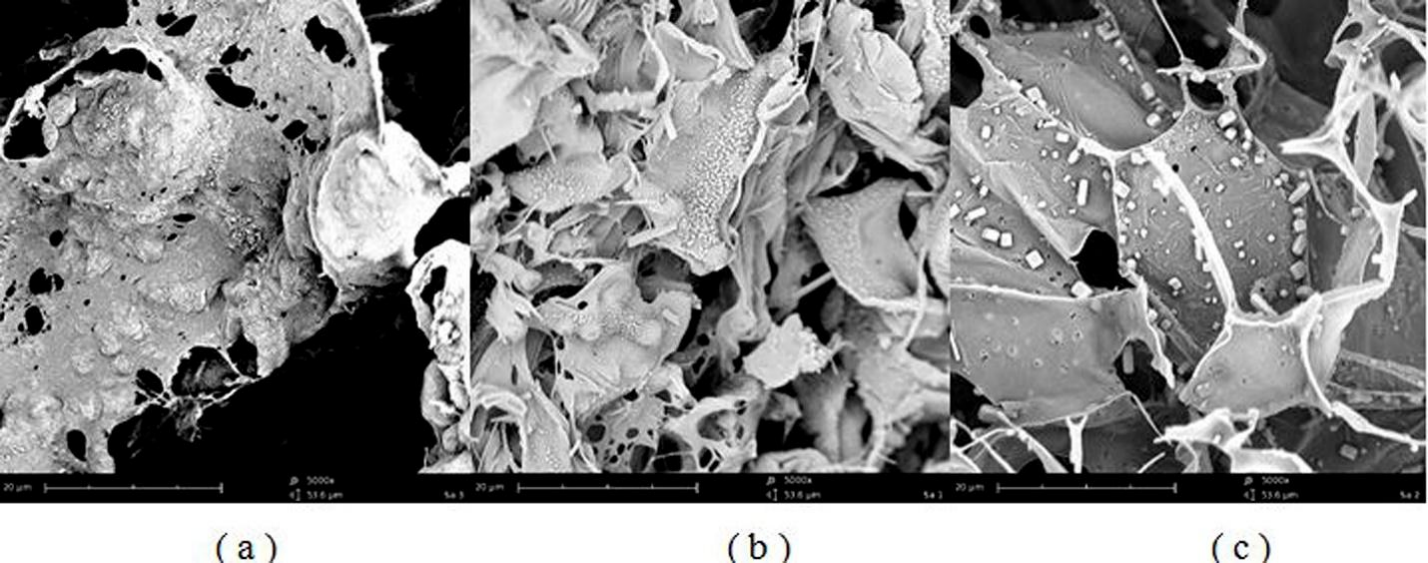
SEM images of spray freeze-dried microparticles: (a) MD1 formulation, (b) L1 formulation, (c) M1 formulation

Scanning electron microscopy of SFD microparticles showed the feather-like particles with porous structure for all of the formulations with no evidence of particle agglomeration. Porous sugars scaffold prevented nanoparticles from aggregation as shown in Figure 5. It was assumed that multiple pores were formed by sublimation of ice crystals. The particles were formed with smooth and shapeless sheet structures. Formulations that contained lactose or maltodextrin as a matrix agent showed uniform particles with no crystals on the surface. However, according to Figure 5c, sugar crystals can be found in formulations containing mannitol.

*Differential scanning calorimetry and FTIR*

**Figure 6:**
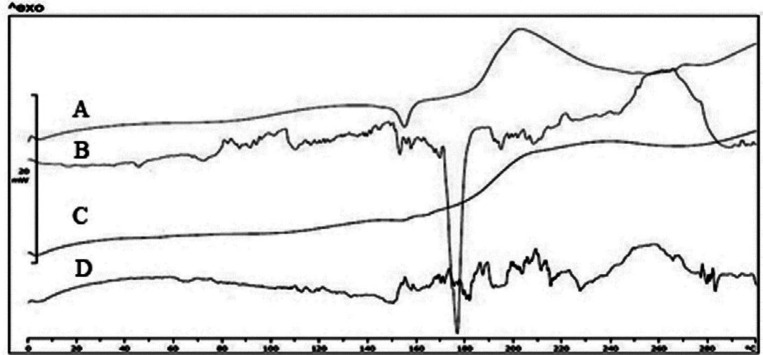
DSC thermograms from top to down is: (A) M1 formulation, (B) pure risperidone, (C) L1 formulation, (D) MD1 formulation

As shown in Figure 6, the pure risperidone exhibited a sharp endothermic peak at 171 ºC corresponding to its melting point which confirmed its crystalline nature. However, thermograms of L1 and MD1 microparticles did not show any distinct peak, which could be due to the amorphous state of risperidone incorporated in particles. The endothermic peak at 152 ºC is related to the crystallization of mannitol in the particles. As mentioned in previous studies, mannitol will form three polymorphisms including α, β, and δ with melting points of 166, 166.5 and 155 ºC, respectively (39). The presence of surfactants and the use of the SFD process led to the formation of δ polymorph (40). The crystal structure was formed when the ratio of mannitol increased. It repeated in spray drying of formulations containing antibody when the mannitol content of formulations exceeded 30% (41). On the other hand, the addition of lactose and maltodextrin did not show any distinct peak due to the formation of amorphous structure in the matrix of the particles. Previous studies showed that the amorphous matrix is appropriate for including extra particles while more crystal regions will push the material out of the matrix (42, 43). Therefore, increasing the amount of mannitol in the formulation will result in the deposition of nanoparticles in the external surface of the microparticles. The results of the DSC tests and SEM images confirmed the crystallinity of mannitol. The mechanical stress caused by the growth of mannitol crystals increased the possibility of nanoparticle aggregation.

Crystallized mannitol can also lead to separation of nanoparticle suspensions and decrease their stability (in comparison to other formulations) (15). Moreover, the formation of mannitol crystals during SFD creates new surfaces. Therefore, surfactant particles can be distributed on new surfaces leading to decreased concentration of surfactants around the nanoparticles and so the probability of nanoparticle growth will increase (31). In parallel, Packhaeuser CB *et al.* showed that mannitol crystallization during freezing drying is responsible for less mannitol protective effect compared to other sugars (44).

The IR spectrum of risperidone was characterized by stretching and bending bands according to Table 3. Risperidone has three stable polymorphs A, B and E. The presence of amide carbonyl-stretching band (at 1650cm^−1^) as a single peak, indicates the presence of the most stable polymorph A in all of the samples (as indicated in DSC results) (45).

**Figure 7:**
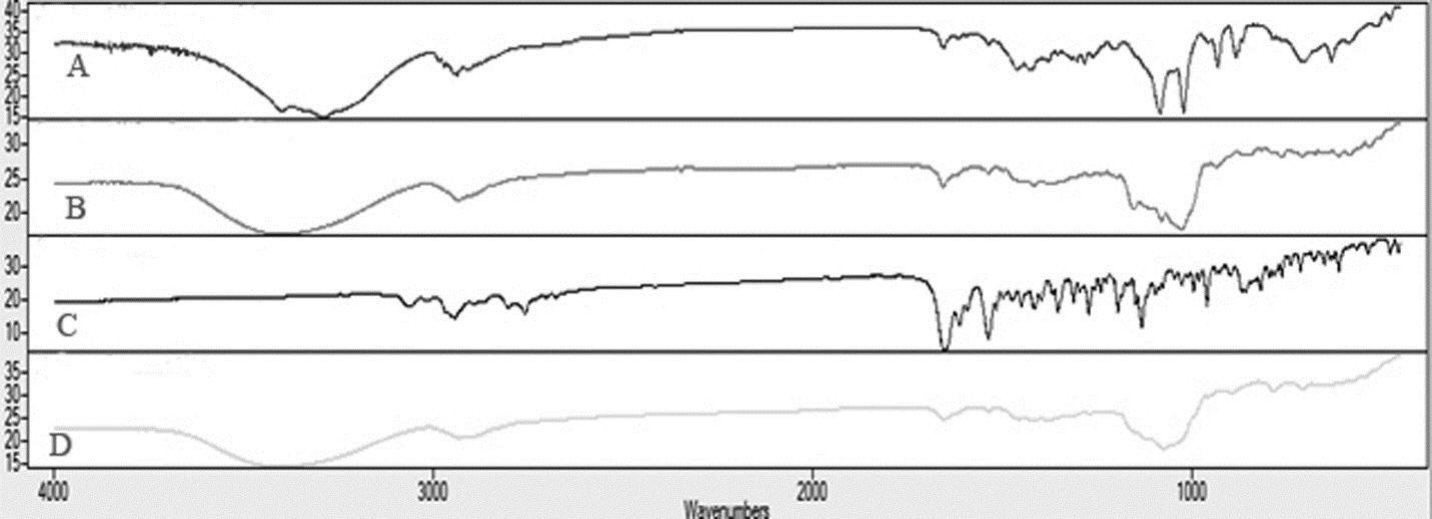
FTIR spectrum from top to down is: (A) 10M formulation, (B) 10MD formulation, (C) pure risperidone and (D) 10L formulation

The appearance of characteristic peaks of risperidone in all of the formulations indicates the absence of chemical interaction between the risperidone and the other excipients, confirming the stability of the drug in nanoparticles (Figure 7).

*Micromeritic properties of microparticles*

The flow properties of microparticles, pure risperidone and nanoparticles were determined by calculating the Carr̕s index and Hausner̕s ratio. As depicted in Table 4, Carr̕s index of pure risperidone was classified as “very poor” flowability because of inter particulate attraction of risperidone coarse particles. Meanwhile, the formulation of microparticles with mannitol at the ratio of 1:1 improved the flowability of powder by decreasing the compressibility index to 29. However, the increase of the mannitol ratio to 5:1 and 10:1 worsen the powder flowability. These results were repeated when lactose and maltodextrin were used in incremental ratio. However, comparing Carr’s indexes showed that the ratio of 1:1 of lactose to risperidone resulted in “good” powder flowability and other formulations showed poor and very poor flowability. It should be noted that the general flowability of porous powder is low, especially in products of SFD with high porosity (3, 46). The proper flowability of the powder will result in the uniformity of the dosage form. Various factors, including morphology of particle, particle size, bulk and tap densities, moisture content, and processing, will affect the flowability of a powder (47). Formulation A with the sticky nanoparticles and lacking matrix agents showed the sticky point as nanoparticles covering with surfactants caused particle adhesion and reduced flowability(14). Matrix agents will increase the flowability by decreasing the adhesion between nanoparticles. Therefore, the increase in the flowability of nanoparticles will be expected in the formulation of microparticles containing matrix agents. Our results indicated that the use of matrix agents (at the ratio of 1:1 to the drug) can improve the flowability of nanoparticles.

*Dissolution study of microparticles and FDT*

**Figure 8:**
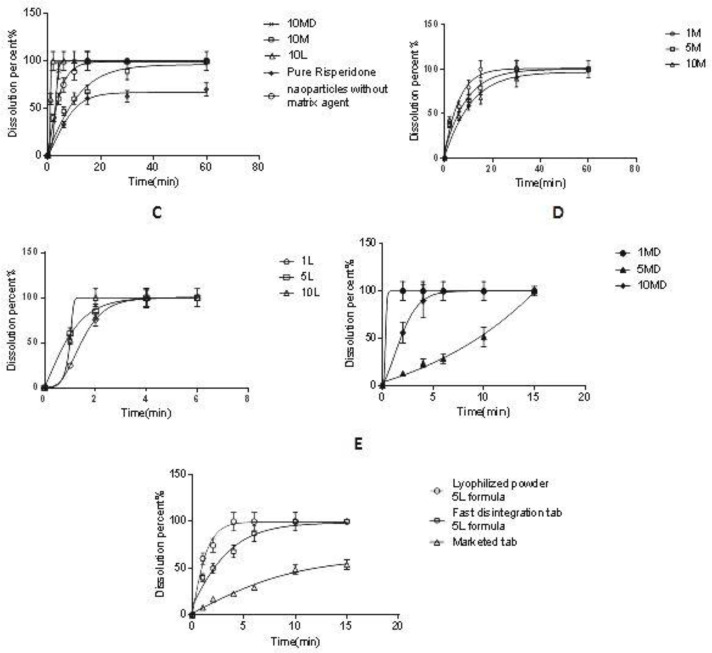
*In-vitro* drug release of: (A) risperidone nanoparticles (10MD, 10M, 10L formula), nanoparticle without matrix former and pure risperidone in SSF, (B) risperidone nanoparticles containing mannitol as a matrix former, (C) risperidone nanoparticles containing lactose as a matrix former, (D) risperidone nanoparticles containing maltodextrin as a matrix former, (E) lyophilized powder of 5L formula, Fast disintegration tab of 5L formula and Marketed tab

The dissolution properties of microparticles were evaluated in 20 mL SSF at 37 ± 0.5 °C. According to Figure 8A, the dissolution rate of the non-processed pure drug was very slow and only 62% of the drug was dissolved after 30 min; however, the selected microparticle (10 L) was dissolved rapidly after 2 min (f2 = 20). The rapid absorption of risperidone in the oral cavity depends on the rapid dissolution of the drug in saliva. However, the data showed that the addition of the carrier to the formulation affected the risperidone dissolution rate. As shown in Figure 8B, the addition of mannitol to the nanosuspension led to a slight retard in the risperidone dissolution. This effect was increased when the mannitol ratio was increased from the ratio of 1:1 to 10:1 (Man/Ris) (f2 = 42.2) and was reversed when lactose was applied as the carrier. As depicted in Figure 8C, the addition of lactose to the microparticles resulted in a higher dissolution rate with a direct relation (f2 = 51.9). However, the addition of maltodextrin showed no predictable effect on the risperidone dissolution rate in all the applied ratios (Figure 8D).

To study the dissolution behavior of risperidone nanoparticles in tablet form, the microparticles were formulated in fast disintegration tablets. Various fast disintegration tablets were developed (data are not shown), and the tablet with including 83.2% MCC, 9.5% SSG and 6% of microparticles (equivalent to 1 mg risperidone) was selected as microparticles carrier with a disintegration time of 13 s. According to the FDA guidance, fast disintegration tablets should be disintegrated within 30 s or less (Food and Administration 2008).

As seen in Figure 8E, the risperidone dissolution of the FDT is slightly slower than SFD risperidone microparticles. However, risperidone was dissolved very quickly from the fast dissolution tablet (10 min) in comparison with the commercial tablet (f2 = 28.5), which was suitable for delivery of risperidone in the oral cavity. According to the Noyes Whitney equation, any reduction in particle size causes an increase in the surface area which has a linear relation with the dissolution rate (48). Also, a decrease in the thickness of the diffusion layer around the particles and an increase of the concentration gradient between the surface of the particle and its surrounding solution can be regarded as other reason (49). On the other hand, the presence of surfactants (with bigger effect) and matrix agents (with lesser effect) can help to increase the dissolution rate. Freeze-drying of nanoparticles which results in the production of highly porous particles with high water penetration efficiency, makes the condition more suitable for hydration. Some articles showed that the use of lactose, as well as other low molecular weight sugars such as trehalose in the tablet matrix, can act as a disintegration agent (50, 51). Therefore, soluble matrix agents such as lactose and maltodextrin will increase the hydration of microparticles and enhance the dissolution rate. However, the results showed that the mannitol (by creating crystals) would worsen the particle agglomeration and lead to a lower dissolution rate compared with lactose and maltodextrin. Indeed, the higher dissolution rate was observed by an increase in the lactose concentration. Higher lactose content will induce a greater reduction in the contact between nanoparticles and hence increase the redispersibility of nanoparticles leading to faster particle hydration and dissolution (15) and this was repeated by using maltodextrin as an oligosaccharide.

**Table 1:** Formulation of risperidone nanosuspension using different stabilizer

**A13**	**A12**	**A11**	**A10**	**A9**	**A8**	**A7**	**A6**	**A5**	**A4**	**A3**	**A2**	**A1**	**Ingredients (w/v%)**
0.2	0.2	0.2	0.2	0.2	0.2	0.2	0.2	0.2	0.2	0.2	0.2	0.2	Risperidone
												0.2	Pvp
											0.2		Hpmc K4M
								0.2					CMC
							0.2						Tween80
0.8	0.6	0.4	0.8	0.6	0.4	0.2							Hpmc E15
0.008	0.008	0.008							0. 008	0.006			Cremophor EL

**Table 2:** Formulation of nanoparticle-containing microparticles of risperidone using different carrier

**Formulation**	**Ingredients (w/v%)**					
	**Risperidone**	**Hpmc E15**	**Cremophor EL**	**Mannitol**	**Lactose**	**Maltodextrin**
A	0. 2	0. 6	0. 008	-	-	-
M1	0. 2	0. 6	0. 008	0. 2	-	-
M5	0. 2	0. 6	0. 008	1	-	-
M10	0. 2	0. 6	0. 008	2	-	-
L1	0. 2	0. 6	0. 008	-	0. 2	-
L5	0. 2	0. 6	0. 008	-	1	-
L10	0. 2	0. 6	0. 008	-	2	-
MD1	0. 2	0. 6	0. 008	-	-	0. 2
MD5	0. 2	0. 6	0. 008	-	-	1
MD10	0. 2	0. 6	0. 008	-	-	2

**Table 3:** Characterization peaks in IR spectrum of risperidone

	**Absorption peak (cm** ^−1^ **)**					
	**1000-1200**	**1129**	**1352**	**1535 and 3060**	**1650**	**3064**
TYPE OF BOND	C–N stretching of the tertiary amine	C-F Stretching of the aryl fluoride	C-N stretching of the oxazole ring	C=C stretching of an arene ring	C=O stretching vibration of the β-lactam ring	C-H asymmetric stretching of aromatic ring

**Table 4.** Micromeritic properties of microparticles

	**1M**	**5M**	**10M**	**1L**	**5L**	**10L**	**1MD**	**5MD**	**10MD**	**A**	**Pure risperidone**
balk density (g/mL)	0. 071	0. 09	0. 06	0. 022	0. 083	0. 05	0. 06	0. 05	0. 05	0. 033	0. 127
tap density (g/mL)	0. 1	0. 15	0. 11	0. 025	0. 1	0. 083	0. 085	0. 1	0. 075	0. 044	0. 212
Carr’s Index	29	40	45. 4	12	17	39. 75	29. 4	50	33. 3	25	40
Hausner’s Ratio	1. 4	1. 66	1. 83	1. 13	1. 2	1. 6	1. 4	2	1. 5	1. 3	1. 66
Flowability	poor	very very poor	very very poor	good	fair	very very poor	poor	very very poor	very poor	passable	very very poor

## Conclusion

Employing Cremophor EL and HPMC E15 as steric stabilizers at the respective concentrations of 0.008% and 0.6% w/v can produce nanosuspension with higher stability. It was indicated that the simultaneous use of polymeric and nonpolymeric surfactants will create a steric barrier around the drug particles and lower the medium surface tension which can lead to the preparation of risperidone nanoparticles. In addition, the dissolution rate of risperidone increased significantly due to the small particle size of nanoparticles according to the Noyes-Whitney equation. Spray freeze-drying was successful in producing dry powder of nanosuspension for preparation of fast dissolving tablet of risperidone with a dissolution time of 10 min.
